# Tracking early cytological changes following expression of NSm and N proteins from tomato spotted wilt virus field isolates

**DOI:** 10.1099/jgv.0.002151

**Published:** 2025-09-12

**Authors:** Alexandra C. Rios, Abdelaal H. A. Shehata, Amanda Strayer-Scherer, Kathleen M. Martin

**Affiliations:** 1 Department of Entomology and Plant Pathology, Auburn University, Auburn, AL, USA; 2 Department of Plant Pathology, Faculty of Agriculture, Assiut University, Assiut, Egypt

**Keywords:** callose deposition, co-localization, non-structural movement protein (NSm), nucleocapsid protein (N), protein localization, tomato spotted wilt virus

## Abstract

Tomato spotted wilt virus (TSWV) is a major yield-limiting pathogen of peanut in the southeastern USA. To assess viral variability, field isolates were collected from symptomatic peanut plants at three locations in Alabama. Sequence analysis identified mutations in the non-structural movement protein (NSm) and the nucleocapsid protein (N). Subcellular localization studies showed that NSm fused to GFP (NSm:GFP) localized to plasmodesmata and co-localized with callose deposits by 2 days post-infiltration (dpi). By 4 dpi, NSm:GFP formed cytoplasmic aggregates, and callose deposition appeared more consistent with basal plasmodesmatal patterns. The N protein localized to the nuclear periphery and cell margins at 2 dpi and later aggregated in the cytoplasm by 4 dpi. The early callose accumulation associated with NSm expression suggests a potential plant defence response, warranting further investigation.

## Data Availability

The TSWV-NSm and N gene sequences used for localization and co-localization in this study are deposited in GenBank with the following accession numbers:

- NSm fused to GFP (N-terminal): BARU16.3-NSm (PP763381), WGREC5.1-NSm (PP779556), GCREC7.2-NSm (PP779557), MT2-NSm (PV841823)
- NSm fused to GFP (C-terminal): BARU16.3-NSm (PV001691), WGREC5.1-NSm (PV001692)
- N fused to GFP or RFP: BARU16.1-N (OR352891), WGREC5.1-N (OR349741), GCREC7.1-N (OR364918), MT2-N (X61799).

## Introduction

Tomato spotted wilt virus (TSWV) (*Orthotospovirus tomatomaculae*) is an ambisense ssRNA member of the order *Elliovirales* [1]. Discovered in Australia in 1915, TSWV has become one of the most economically devastating viruses worldwide [1]. It was first identified in the USA in Texas in 1971 and has since spread throughout the southeastern region, causing several severe outbreaks in important hosts such as tomatoes and peanuts [2]. Despite the introduction of resistant peanut cultivars [3,5], TSWV remains a challenge. The genome of TSWV consists of three RNA segments, known as the large (L), medium (M) and small (S) genomic RNA, all contained within a spherical envelope [6]. The L RNA encodes the RNA-dependent RNA polymerase (L), the M segment encodes the non-structural medium component protein (NSm, movement) and the precursor of the glycoproteins (G_N_ and G_C_). Lastly, the S segment encodes the non-structural small component protein (NSs, silencing) and the nucleocapsid (N) [6].

At the onset of these experiments, a previous study focused on field mutations found in the TSWV nucleocapsid protein, noting mutations [7], leading to the expectation that mutations could also be found in other genes, like NSm, which has been shown to interact with N in some strains [8,9]. Previous data on TSWV-MT2 NSm indicated that it moved from the cell periphery to more central regions from days 2 to 3 [10], and N also changed its localization pattern between days 2 and 8 [10,11]. This study focused on the NSm and the N in field strains. NSm is the putative movement protein, forming tubular structures within the plasmodesmata of the plant cell, allowing for cell-to-cell movement of the ribonucleoprotein complex [12]. In contrast, N is involved in viral gene transcription and genome replication and binds to the genomic RNA to form ribonucleoprotein complexes [13,15]. While previous studies have characterized TSWV strains such as MT2 [10] and L3 [9], little is known about the localization and co-localization behaviours of field-collected strains. This study investigates the NSm and N proteins from TSWV field isolates, comparing their sequence variation and subcellular localization with those of a well-studied laboratory strain [10]. The objective was to assess whether mutations in field isolates affect protein trafficking and co-localization, which may explain variation in host interactions and adaptation.

## Methods

### Field sample collection, RNA extraction and N/NSm amplification

Symptomatic peanut plants were collected from three locations in Alabama, including Brewton Agricultural Research Center (BARU) in Brewton, AL, Wiregrass Research and Extension Center (WGREC) in Headland, AL, and Gulf Coast Research and Education Center (GCREC) in Fairhope, AL, in 2022. RNA extraction of the leaves was performed [16]. Both the forward NSm primer (5′ CACCATGTTGACTCTTTTCGGTAATAAGG 3′) and the reverse NSm primer (5′ TTATATTTCATCAAAAGACAACTGAGCAACACTG 3′) were used for amplification using the following settings: 95 °C–2 m; 34X: (95 °C–1 m, 57 °C–1 m, 72 °C–45 s); 72 °C–5 m and hold at 12 °C using the Phusion High-Fidelity Polymerase (Thermo Fisher Scientific: F530S) [10]. One sample from each field, BARU16.3, WGREC 5.1 and GCREC7.2, was also amplified using a non-stop reverse NSm primer (5′ TATTTCATCAAAAGATACTGAGCAAC 3′) [10]. This produced three constructs that contained stop codons and three that did not, which were used in pENTR-d-TOPO reactions following the manufacturer’s directions (Invitrogen, Carlsbad, CA). The matching N sequences represent the three locations and were amplified via PCR as previously described [10].

### Sequence analysis and construction of the phylogenetic tree

The sequences were verified and analysed using National Center for Biotechnology Information (NCBI) blast [17], translated from DNA to protein sequence, and aligned against 18 other closely related TSWV-NSm sequences from Florida (AY956380.1, KU179544.1, KU179542.1, KU179550.1 and KU179546.1), Georgia (AY870390.1, KU179600.1 and KU179616.1), North Carolina (AY744487.1, AY744489.1, AY744488.1, KU179528.1 and OP832374.1, and AY744490.1), South Carolina (KU179592.1, KU179516.1 and KU179598.1) and Virginia (KU179524.1) to map their mutations [18]. A maximum likelihood phylogenetic tree was created from the constructed alignment using mega11 [19], as described previously [16]. The phylogenetic tree was constructed with 1,000 bootstraps and exported from mega11 in Newick format and visualized using the Interactive Tree of Life (iTOL) (https://itol.embl.de/tree/13120425486293691674071207) [20].

### Construct design, infiltration of plants and microscopy

The MT2 strain of TSWV, originally isolated from Hawaii and representative of U.S. isolates [11], was used as a control in this study. Entry clones of NSm were recombined into vectors containing either GFP fusions in either N-terminal (pSITE-2-NA) or C-terminal (pSITE-2-CA) orientations, as previously described [10,16, 21]. Constructs were transformed into *Agrobacterium tumefaciens* and infiltrated into WT *Nicotiana benthamiana* leaves, following established protocols [16]. N sequences from field isolates BARU16.1 (OR352891), WGREC5.1 (OR349741) and GCREC7.1 (OR364919) were fused to the N-terminus of GFP (pSITE-2-NA) for localization studies and to the N-terminus of RFP (pSITE-4-NA) for co-localization assays with NSm. *Agrobacterium* containing constructs intended for co-expression were mixed at a 1 : 1 ratio prior to infiltration and introduced into WT *N. benthamiana* leaves as previously described [16].

For subcellular visualization, leaves infiltrated with NSm-GFP or GFP-NSm constructs were stained with aniline blue fluorochrome (Thermo Fisher Scientific, Cat# 189350250) to label plasmodesmata (PD), as previously described [18], with an increased concentration by a factor of 10 due to difficulty in visualizing the PD during imaging. Leaves expressing N-RFP were stained with 10 ng µl^−1^ DAPI (Thermo Fisher Scientific, Cat# 62248) for 30 min prior to imaging to visualize nuclei. As a negative control for localization, pSITE-2CA was used, containing GFP [11,13]. Imaging was performed at 2-, 3- and 4 days post-infiltration using a Nikon Eclipse Ts2R epifluorescence microscope at 40× magnification with NIS-Elements Viewer software. For each of the three biological replicates, images of at least three plant cells were collected. A fourth replicate was imaged using confocal microscopy, as previously described [16]. Final image panels were assembled using Microsoft PowerPoint.

### Immunoblotting

Co-infiltrated leaf tissues with NSm-GFP and N-RFP from 2 and 5 days post-infiltration (dpi) for two replicates were stored at −20 °C in Laemmli buffer for use in SDS-PAGE and immunoblotting. To detect NSm-GFP expression, rabbit anti-GFP (BIO-RAD Ref: S373B) was used as the primary antibody, and goat anti-rabbit (BIO-RAD Ref: 12004161) was used as the secondary antibody. Transgenic *N. benthamiana* plants expressing GFP-ER as a positive control, while WT *N. benthamiana* leaf tissues were used as a negative control.

## Results and discussion

### NSm sequence variation highlights distinct mutation patterns among Alabama isolates

**Fig. 1.**
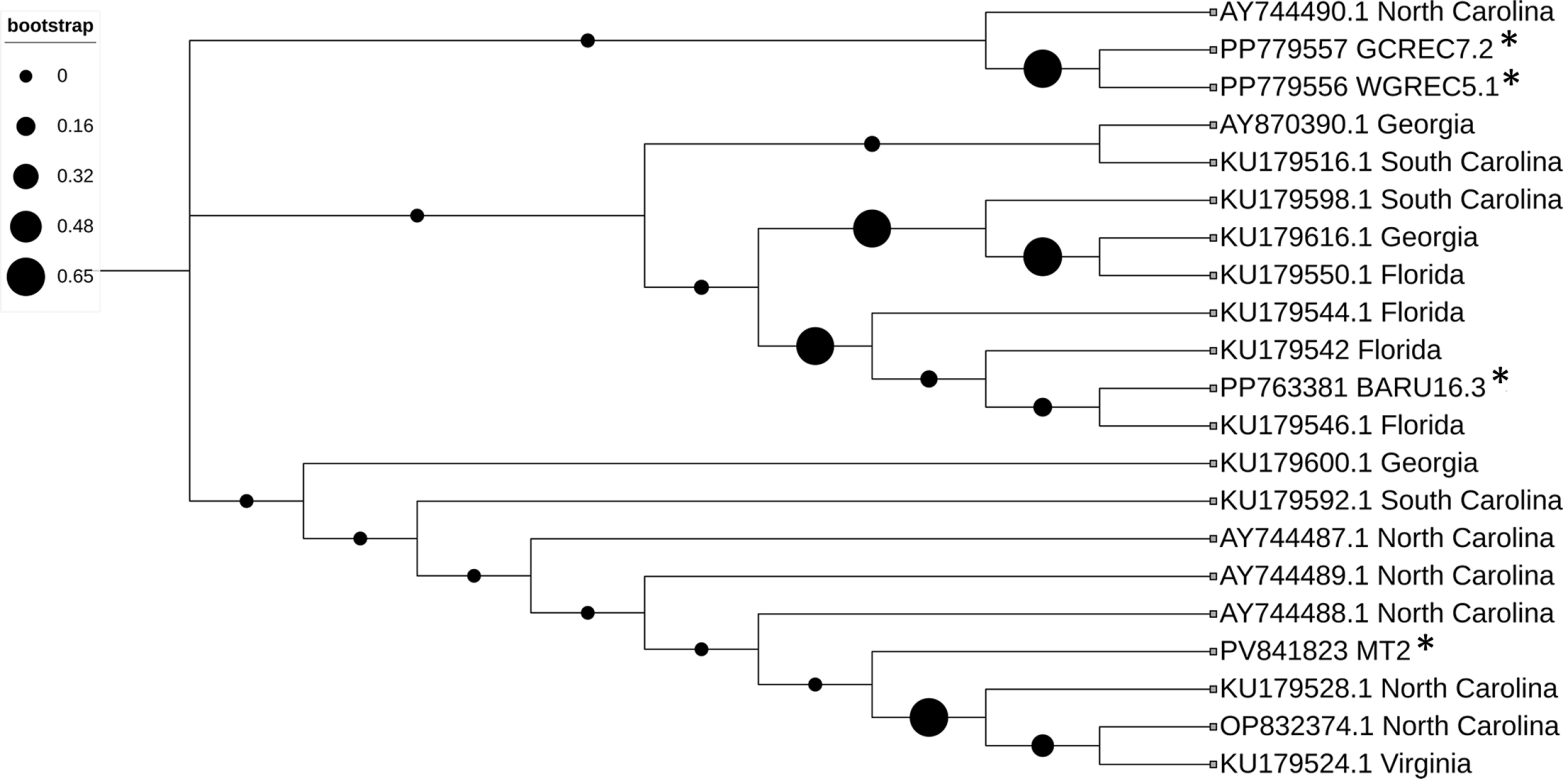
Maximum likelihood phylogenetic tree based on the alignment of 302 amino acids of the NSm protein from 21 TSWV isolates, including 3 Alabama (AL) isolates characterized in this study, the MT2-NSm strain, as well as 17 other strains of TSWV-NSm from other states to AL from the GenBank database. Sequences were aligned using muscle, and the phylogenetic tree was constructed in mega11 using the maximum likelihood method with 1,000 bootstrap replicates. The tree is unrooted and was visualized using the iTOL. Bootstrap support values are shown at each branch. Asterisks (*) denote the AL isolates that were localized in this study. The MT2-NSm sequence is included in the analysis.

Phylogenetic analysis (Fig. 1) shows WGREC5.1 and GCREC7.2 are closely related, differing by two amino acid changes in NSm at position 288: WGREC5.1 encodes a glycine–arginine (GR) motif, while GCREC7.2 has glutamic acid–glycine (EG). BARU16.3 clusters with three Florida isolates, differing from WGREC5.1 by six amino acid changes across NSm. No conserved changes were found across all three Alabama isolates and the NSm-MT2 strain, indicating NSm variability. BARU16.3 has two unique N-terminus substitutions (L44F and A49V), both hydrophobic. While WGREC5.1 and GCREC7.2 share a single C-terminus change (L290T), which altered a hydrophobic to polar uncharged residue, the only common change is unexpected as position 290 is not linked to any known NSm functional domain [12,22].

The conserved L290T substitution among WGREC5.1 and GCREC7.2 is notable. These isolates are also highly similar to their matching *N* gene, differing only at position 212, where a conservative amino acid substitution (arginine to lysine) is observed, agreeing with previous results that *N* is the least diverse in southern isolates [23]. Their limited variation suggests a close evolutionary relationship and recent common ancestry. BARU16.3, which is more closely related to Florida isolates and has divergent N-terminal NSm changes, lacks the L290T substitution altogether. These differences suggest that the three isolates may be evolving under distinct selective pressures, resulting in unique mutations. Although the functional effects of these mutations are unknown, the consistent L290T change in two distinct isolates suggests a potential site for further study.

### NSm and N localization reveal time-dependent aggregation and callose association

**Fig. 2.**
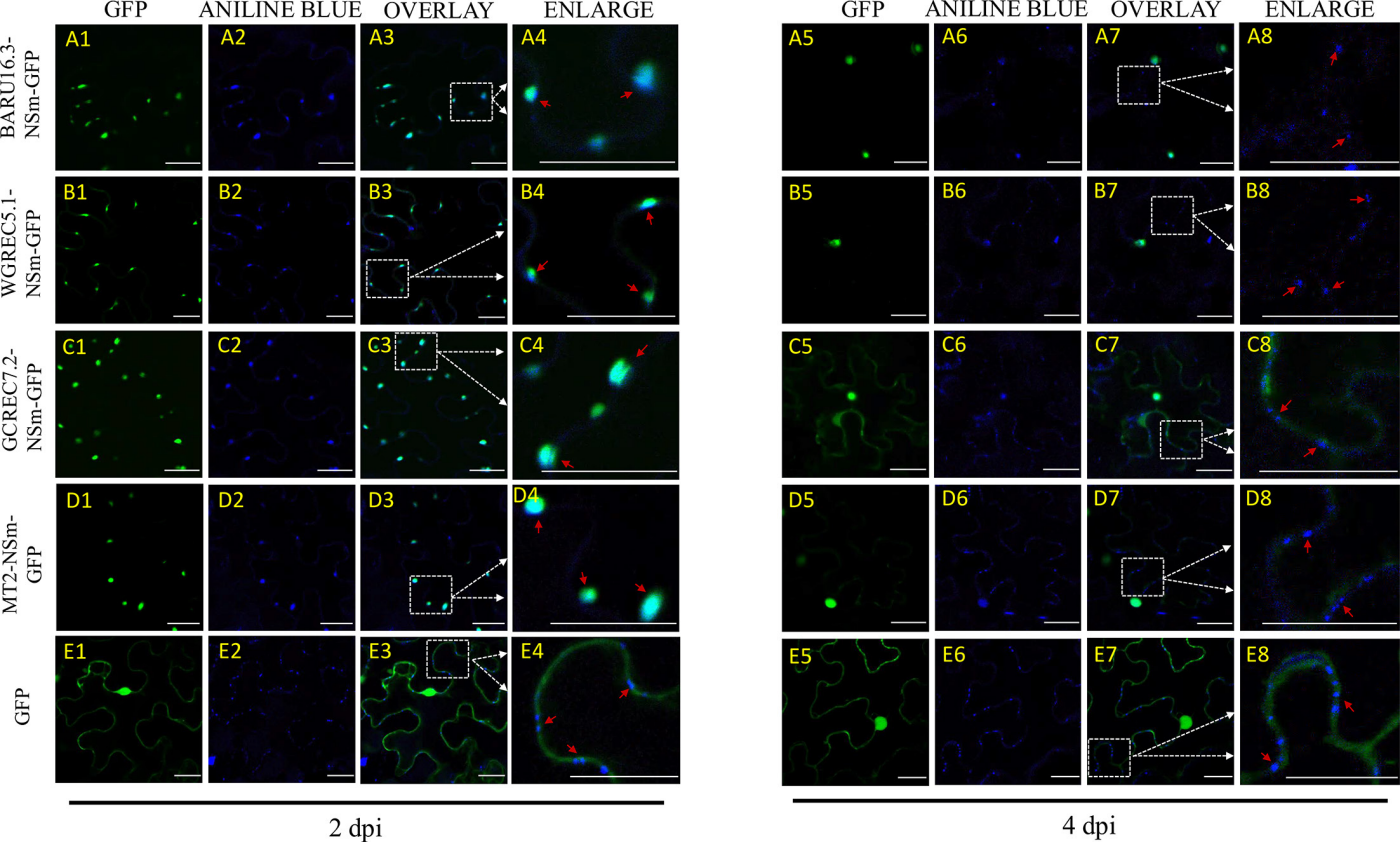
Confocal microscopy images of the localization of NSm:GFP in the plant cell epidermal of the WT *N. benthamiana*. The block on the left shows the images taken at 2 dpi, and the right block shows the images taken at 4 dpi. In both blocks, columns from left to right are TSWV-NSm tagged to the N terminus of GFP, aniline blue stain, an overlay between columns one and two and an enlarged section of the overlayed images highlighted with dotted boxes. From top to bottom, the rows are (**a1–a8**) BARU16.3-NSm:GFP (PP763381), (**b1–b8**) WGREC5.1-NSm:GFP (PP779556), (**c1–c8**) GCREC7.2-NSm:GFP (PP779557), (**d1–d8**) MT2-NSm:GFP (PV841823) and (**e1–e8**) Free GFP. Arrows are pointing to the difference observed in the PD size between the localized NSm isolates and the control free-GFP between 2 and 4 dpi. The scale bar is 25µm.

Localization studies examined Alabama isolates’ NSm and N proteins’ subcellular distribution via GFP fusion in *N. benthamiana*. To capture early localization while minimizing toxicity, which can occur with viral proteins [16], imaging was performed at 2 and 4 dpi, when GFP is typically detectable (Fig. 2). In prior work, localization of TSWV NSm (MT2) was tracked for 3 days, showing peripheral localization at 2 dpi and a shift to more central areas by 3 dpi, without observed toxicity [10]. N localization has been observed for up to 8 dpi, but changes largely occur early, with later stages dominated by aggregation without significant positional shifts [11]. In these experiments, aniline blue was used to stain callose as a marker for plasmodesmata, providing spatial context for NSm localization. Aniline blue, used in capsicum chlorosis virus (CaCV) and other tospoviruses [24,25], binds callose, a *β*-1,3-glucan, at plasmodesmata, especially during stress and defence responses [26,28]. This study used aniline blue to stain callose at plasmodesmata, marking NSm localization. The staining reflects either plasmodesmata structure or a host response, enabling assessment of NSm’s potential role in callose deposition.

At two dpi, NSm-GFP (N-terminal fusions) localized strongly to the cell periphery and co-localized with aniline blue, suggesting association with plasmodesmata (Fig. 2a1–d1). By 4 dpi, NSm-GFP formed cytoplasmic aggregates, accompanied by a decrease in peripheral signal. Leaves expressing NSm-GFP showed greater aniline blue signal at 2 dpi than the free-GFP controls (Fig. 2a4–d4 and e4). This elevated signal diminished by 4 dpi, becoming punctate and more restricted to plasmodesmatal necks [18]. GFP:NSm (C-terminal fusions) followed distinct kinetics seen at 2 dpi when both BARU16.3 and WGREC5.1 formed cytoplasmic aggregates with no PD localization recorded (Fig. S1A1–A3, B1–B3, available in the online Supplementary Material). By 4 dpi, GFP:NSm-WGREC5.1 redistributed toward the periphery, while GFP:NSm-BARU16.3 remained aggregated (Fig. S1A4–A6, B4–B6). GCREC7.2 could not be analysed due to fusion instability. The patterns observed in this study, based on C-terminal GFP:NSm fusions, are consistent with the idea that C-terminal fusion could disrupt important motifs on the N-terminal of NSm, such as the tubule formation domain or the conserved P/D-l-X motif, potentially altering localization. Similar orientation-dependent effects have been reported for NSm in SVNV [16], highlighting the importance of careful fusion design in localization studies. N-GFP constructs localized near the nucleus and at the cell periphery at 2 dpi (Fig. S2A1–D3), but by 4 dpi, transitioned to diffuse cytoplasmic aggregates (Fig. S2A4–D4). Among isolates, GCREC7.1-N-GFP showed more pronounced and earlier aggregation, resembling the MT2 control pattern [11,19] (Fig. S2C1 and C6).

Taken together, these results show that NSm initially localizes to plasmodesmata, consistent with its role as a movement protein [8,10, 22], and correlates with a transient increase in callose accumulation. By 4 dpi, both NSm localization and callose signal diminish at the cell periphery. This pattern, absent in CaCV [18], suggests virus-specific interactions with host structures. Callose itself does not relocalize but is deposited by plasmodesmatal callose synthases and removed by *β*-1,3-glucanases [29,30]. The early increase in aniline blue staining likely reflects NSm-induced callose synthesis via defence signalling or direct structural effects. As infection progresses, NSm may no longer stimulate this response or may actively promote callose turnover to enable movement, consistent with the dynamic, reversible regulation of callose during viral spread [29,31].

### Isolate-specific differences in NSm–N colocalization reveal distinct patterns of aggregation

TSWV N and NSm proteins play central roles in viral replication and cell-to-cell movement [8,9, 12, 14]. Yet, their functional relationship – especially in natural field variants – remains poorly understood. NSm traffics viral RNPs [8], while N encapsidates the genome and may be co-transported [9]. Prior studies have suggested that N and NSm may or may not physically interact, depending on the viral strain [9,10], raising important questions about whether their intracellular colocalization is consistent across isolates and what functional differences might arise from strain-specific variation. Investigating N–NSm colocalization provides insight into the molecular coordination of replication and movement and may reveal isolate-specific adaptations that impact virus spread or host interaction.

**Fig. 3.**
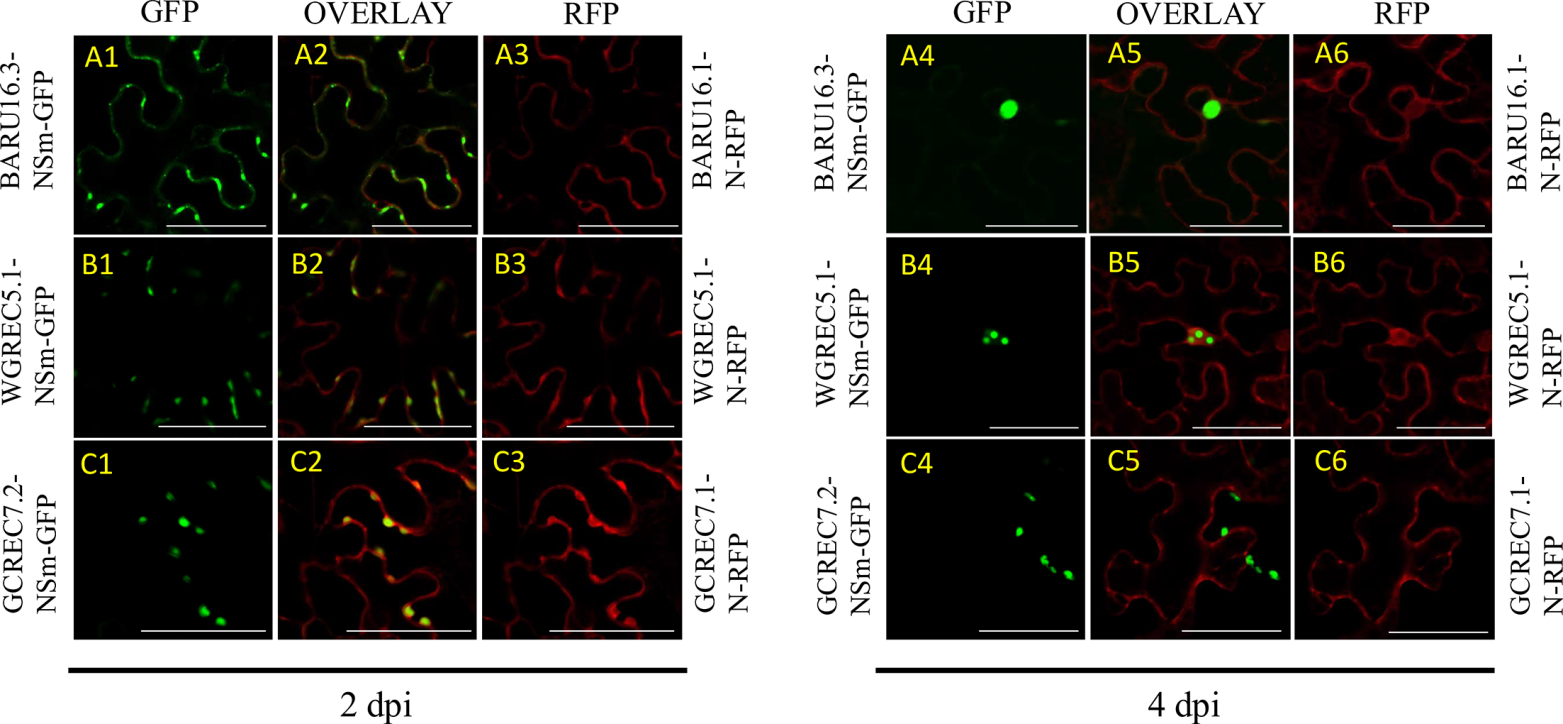
Confocal microscopy images of the co-localization of NSm:GFP and N:RFP in the plant cell epidermis of the WT *N. benthamiana*. The block on the left shows the images taken at 2 dpi, and the right block shows the images taken at 4 dpi. In both blocks, columns from left to right are TSWV-NSm tagged in the N terminal of GFP, an overlay between columns one and three and TSWV-N tagged in the N terminal of GFP. From top to bottom, the rows are (**a1–a6**) BARU16.3-NSm:GFP (PP763381) with BARU16.1-N:RFP (OR352891), (**b1–b6**) WGREC5.1-NSm:GFP (PP779556) with WGREC5.1-N:RFP (OR349741) and (**c1–c6**) GCREC7.2-NSm:GFP (PP779557) with GCREC7.1-N:RFP (OR364918). The scale bar is 50 µm.

Fluorescent microscopy of coexpressed NSm:GFP and N:RFP in plant cells revealed both conserved and divergent colocalization patterns among three field isolates – BARU16.3-NSm and BARU16.1-N, WGREC5.1-NSm and WGREC5.1-N, and GCREC7.2-NSm and GCREC7.1-N (Fig. 3a1–c6). At 2 dpi, all isolates showed clear colocalization of N and NSm, indicating early-stage coordination of these proteins across variants. However, by 4 dpi, distinct isolate-specific differences emerged in both aggregation and distribution. In BARU16.3, NSm:GFP formed a large, condensed intracellular aggregate, while N:RFP remained diffusely localized at the cell periphery and did not appear to aggregate (Fig. 3a4–a6). In WGREC5.1, NSm:GFP aggregated into smaller clusters compared to BARU16.3, and N:RFP was also found at the periphery, partially surrounding the NSm clusters (Fig. 3b4–b6). GCREC7.2 exhibited a similar pattern to WGREC5.1, though its NSm aggregates were more evenly dispersed throughout the cytoplasm, and N:RFP remained primarily peripheral and soluble (Fig. 3c4–c6). These data show that, although NSm and N colocalize at early stages of infection, their later spatial organization and aggregation behaviour vary by isolate.

N consistently remains peripheral in the co-localization studies, while NSm becomes increasingly aggregated over time, suggesting differential mobility or compartmentalization. When compared to N:GFP expressed alone, which begins at the cell periphery and later forms central aggregates (Figs 3 and S2), this pattern is consistent with earlier reports on TSWV-N [10,11]. Those studies also noted that aggregate GFP signal intensity can overshadow the relatively weak peripheral localization, making N appear more centralized compared to RFP fusions (Fig. 3) [10,21]. While this study does not establish whether N and NSm interact directly in these isolates, the progression from early colocalization to what appears as distinct localization patterns may partially reflect differences in fluorophore intensity and detection, rather than complete uncoupling. These observations suggest coordinated localization, influenced by sequence-specific factors, but confirmation needs further study.

Immunoblotting confirmed the expression and relative stability of the fusion proteins (Fig. S3). Bands corresponding to GFP (~26.95 kDa) and NSm-GFP (~60.75 kDa) were detected in all three field isolates, while no signal was observed in WT plant extracts. Additional faint bands between 26 and 59 kDa may reflect partial degradation or post-translational modifications, possibly due to sample handling and storage conditions. These results indicate the expression of the fusion protein, supporting the microscopy findings.

In summary, sequence, localization and co-localization analyses reveal both conserved roles and isolate-specific differences in NSm and N across TSWV field strains. NSm variation, such as the unique BARU16.3 substitutions and the shared L290T in WGREC5.1 and GCREC7.2, reflects ongoing evolution. NSm consistently targets plasmodesmata early and then shifts to cytoplasmic aggregation as callose association declines. N follows a different localization path, becoming increasingly aggregated. Though NSm and N initially colocalize, distinct patterns emerge by 4 dpi, indicating isolate-dependent localization dynamics. These findings suggest that temporally regulated NSm–N coordination, shaped by sequence variation, provides a foundation for exploring how natural viral diversity impacts infection and host interactions.

## Supplementary material

10.1099/jgv.0.002151 Uncited Supplementary Material 1.

## Abbreviations

BARU: Brewton Agricultural Research Center
CaCV: capsicum chlorosis virus
dpi: days post-infiltration
GCREC: Gulf Coast Research and Education Center
iTOL: Interactive Tree of Life
L: large
M: medium
N: nucleocapsid protein
NSm: non-structural movement protein
PD: plasmodesmata
S: small
TSWV: tomato spotted wilt virus
WGREC: Wiregrass Research and Extension Center
