# Supplementary material for: Tracking early cytological changes following expression of NSm and N proteins from tomato spotted wilt virus field isolates
Source: J Gen Virol. 2025 Sep 12;106(9):002151. doi: 10.1099/jgv.0.002151 (PMC12451621; doi:10.1099/jgv.0.002151)
Supplement: Uncited Supplementary Material 1. [file jgv-106-02151-s001.pdf]

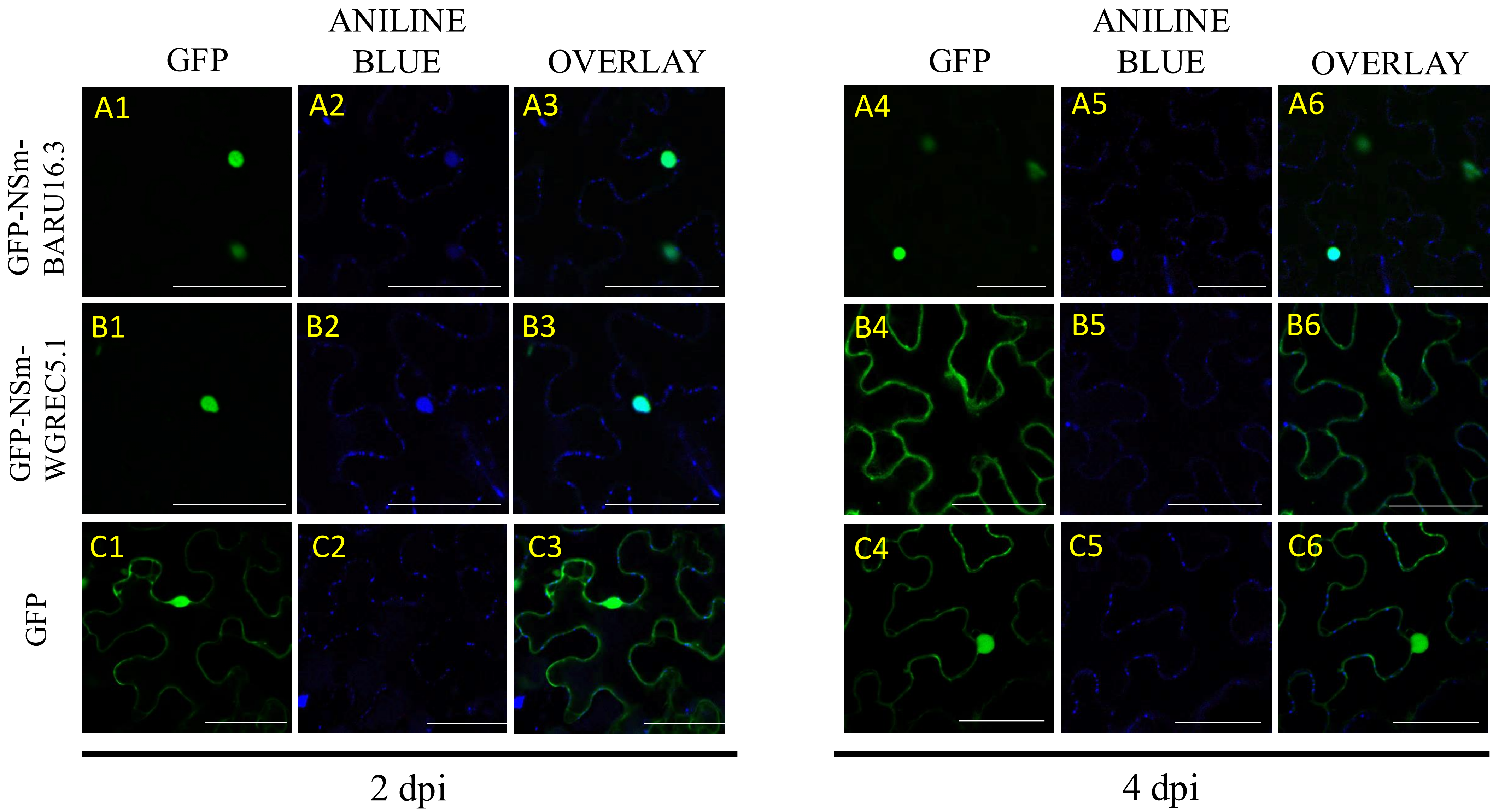

Supplementary Figure 1. Confocal microscopy images of the localization of GFP:NSm in the plant cell epidermal of the WT *N. benthamiana*. The block on the left shows the images taken at two days post-infiltration (dpi) and the right block shows the images taken at four dpi. In both blocks, columns from left to right are: TSWV-NSm tagged in the C-terminal of GFP, aniline blue stain, and an overlay between columns one and two. From top to bottom, the rows are (A1-A6) GFP:BARU16.3-NSm (PV001691), (B1-B6) GFP:WGREC5.1-NSm (PV001692), and (C1-C6) Free GFP. The scale bar is 50 μm.

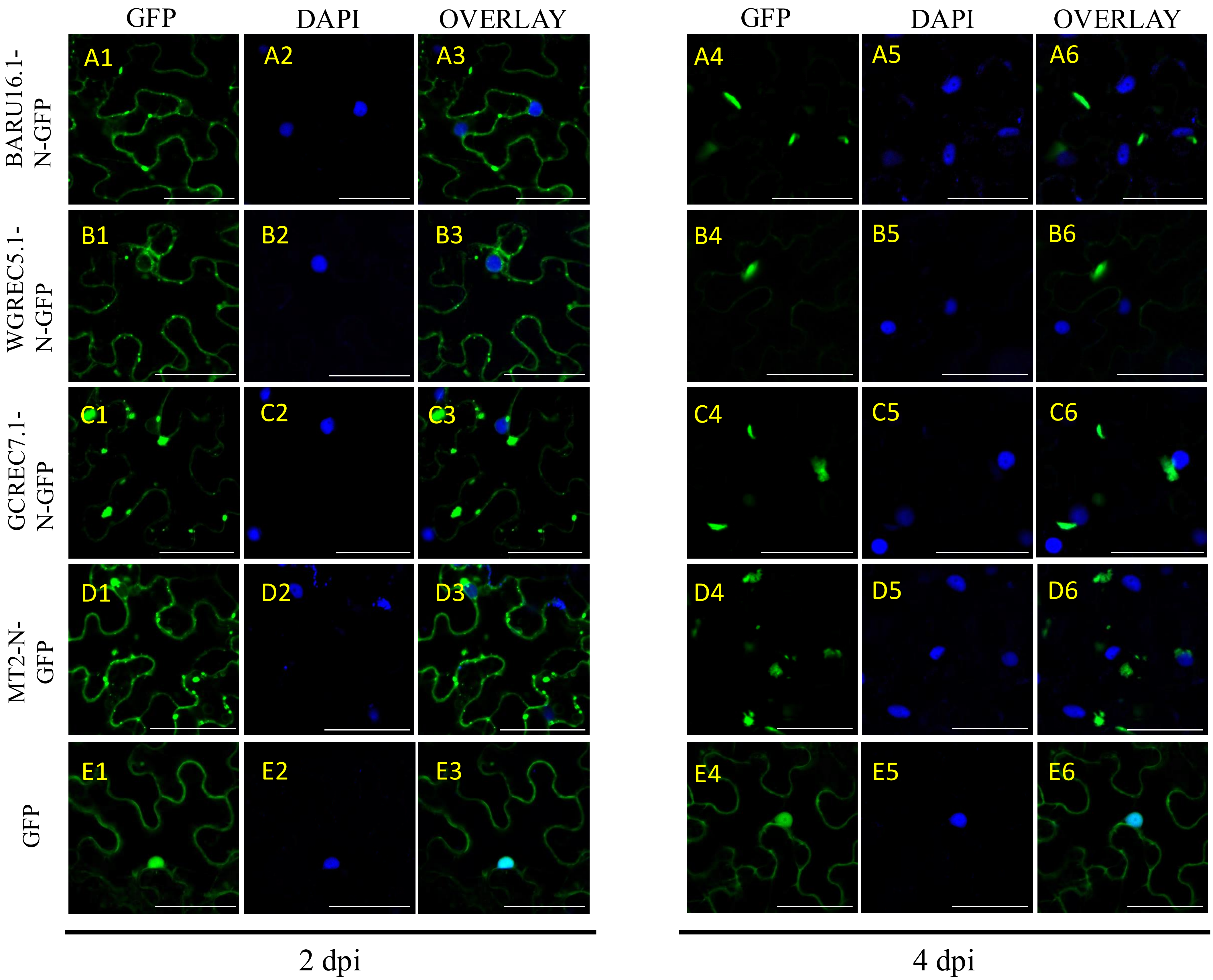

Supplementary Figure 2. Confocal microscopy images of the localization of N: GFP in the plant cell epidermal of the WT *N. benthamiana*. The block on the left shows the images taken at two days post-infiltration (dpi), and the right block shows the images taken at four dpi. In both blocks, columns from left to right are: TSWV-N tagged in the N terminal of GFP, DAPI stain, and an overlay between columns one and two. From top to bottom, the rows are (A1-A6) BARU16.1-N:GFP (OR352891), (B1-B6) WGREC5.1-N:GFP (OR349741), (C1-C6) GCREC7.1-N:GFP (OR364918), (D1-D6) (MT2) MT2-N:GFP (X61799), and (E1-E6) Free GFP. The scale bar is 50 μm.

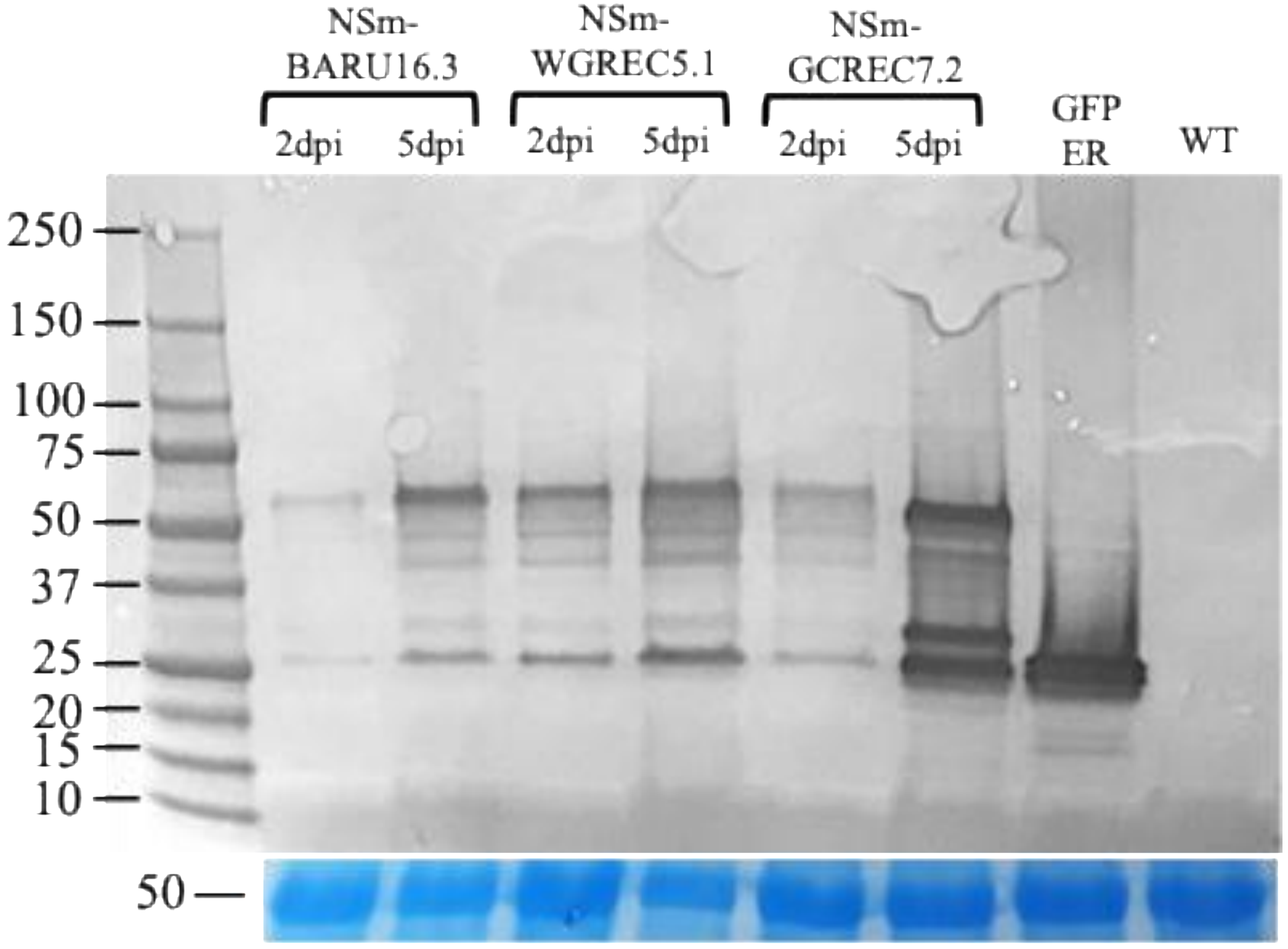

Supplementary Figure 3. Validation of the protein expression of the co-localization of NSm-GFP (N-terminal) with N-RFP (N-terminal) by western blot. The top image depicts the immunoblot, and the bottom image depicts the gel stained with Coomassie Brilliant Blue SDS-PAGE gel, which shows equal loading of all tested samples and is marked at 50 kDa. The protein being detected in this western blot is NSm fused to the N-terminal of GFP (NSm-GFP). NSm alone is ~33.8 KDA, and GFP alone is ~26.95 KDA; infusion of NSm-GFP is ~60.75 KDA, which appears slightly above the 50 KDA band on the ladder. From left to right, the top image has a 1 Kb molecular weight marker. From left to right, the two images have BARU16.3-NSm:GFP at two dpi, BARU16.3-NSm:GFP at five dpi, WGREC5.1-NSm:GFP at two dpi, WGREC5.1-NSm:GFP at five dpi, GCREC7.2-NSm:GFP at two dpi, GCREC7.2-NSm:GFP at five dpi, two spacer wells, GFP-ER, and wild-type (WT).
